# DMXL2 drives epithelial to mesenchymal transition in hormonal therapy resistant breast cancer through notch hyper-activation

**DOI:** 10.18632/oncotarget.4164

**Published:** 2015-06-04

**Authors:** Monica Faronato, Van T.M. Nguyen, Darren K. Patten, Ylenia Lombardo, Jennifer H. Steel, Naina Patel, Laura Woodley, Sami Shousha, Giancarlo Pruneri, R. Charles Coombes, Luca Magnani

**Affiliations:** ^1^ Department of Surgery and Cancer, Imperial College London, London, UK; ^2^ Division of Pathology, European Institute of Oncology and University of Milan, School of Medicine, London, UK

**Keywords:** DMXL2, breast cancer, notch, endocrine therapy, EMT

## Abstract

The acquisition of endocrine therapy resistance in estrogen receptor α (ERα) breast cancer patients represents a major clinical problem. Notch signalling has been extensively linked to breast cancer especially in patients who fail to respond to endocrine therapy. Following activation, Notch intracellular domain is released and enters the nucleus where activates transcription of target genes. The numerous steps that cascade after activation of the receptor complicate using Notch as biomarker. Hence, this warrants the development of reliable indicators of Notch activity. DMXL2 is a novel regulator of Notch signalling not yet investigated in breast cancer. Here, we demonstrate that DMXL2 is overexpressed in a subset of endocrine therapy resistant breast cancer cell lines where it promotes epithelial to mesenchymal transition through hyper-activation of Notch signalling via V-ATPase dependent acidification. Following DMXL2 depletion or treatment with Bafilomycin A1, both EMT targets and Notch signalling pathway significantly decrease. We show for the first time that DMXL2 protein levels are significantly increased in ERα positive breast cancer patients that progress after endocrine therapy. Finally, we demonstrate that DMXL2 is a transmembrane protein with a potential extra-cellular domain. These findings identify DMXL2 as a novel, functional biomarker for ERα positive breast cancer.

## INTRODUCTION

Notch signalling is frequently deregulated in estrogen receptor positive breast cancer and represents a challenging but important therapeutic opportunity [1]. At least in some respect, difficulties in targeting Notch arise from the complex molecular features of this pathway [2]. In mammals, there are four Notch receptors and five Notch ligands. The activity of both is regulated by endocytic trafficking, although the detailed mechanisms are yet to be fully defined [3]. Data suggest that defective endosomes maturation could cause aberrant Notch signalling by interfering with γ-secretase (GS) mediated Notch cleavage [4]. At present, Notch can be targeted in the clinic with γ-secretase inhibitors (GSI), a series of compounds designed to block Notch cleavage and nuclear translocation of Notch intracellular domain. Furthermore, antibodies against Notch are currently being tested [5] although patients reported side effects similar to the ones due to the use of GSIs [6]. Notch plays a pivotal role in many processes including embryonic development, carcinogenesis, cancer stem cells maintenance, angiogenesis, and epithelial to mesenchymal transition (EMT) [7–13].

Aberrant Notch signalling has been linked to breast cancer formation and progression [14]. Breast cancer remains a major clinical problem worldwide, accounting for over for 23% of all cancer deaths [15]. Nearly 70% of breast cancers express estrogen receptor α (ERα) and are treated with adjuvant endocrine therapy designed to block ERα driven transcriptional programs. Alas, around 40% of patients evolve resistance to endocrine therapies and progress towards more advanced disease [16–18]. The mechanisms behind drug resistance have not yet been fully elucidated, although work from our and other groups proposes that epigenetic reprogramming leading to increased Notch signalling may contribute to it [19–21]. Notch involvement in endocrine therapy resistance makes it attractive for developing targeted therapies. Thus, there is a strong need for Notch based biomarkers, especially with potential applicability for clinical assays. This prompted us to investigate DMXL2, which was recently presented as a novel Notch modulator. The *DMXL2* gene has been recently described as a novel player in Notch signalling, regulating the acidification of intracellular compartments through the vacuolar protonic pump (V-ATPase) both in Drosophila and in mammalian systems [22, 23]. The V-ATPase pump plays an important role in vesicular trafficking alongside endocytic and exocytic traffic [24]. We investigated the role of DMXL2 in the context of endocrine therapy resistance using patients' derived clinical specimens and long-term estrogen deprived LTED cells [25–28]. Our results demonstrate that DMXL2 regulates Notch cleavage and chromatin recruitment, epithelial to mesenchymal transition, invasion and migration of endocrine therapy resistant breast cancer cells. Hence, DMXL2 can be identified as novel biomarker in ERα positive breast cancer patients.

## RESULTS

### Notch signalling is dependent on notch accumulation in the chromatin

**Figure 1:**
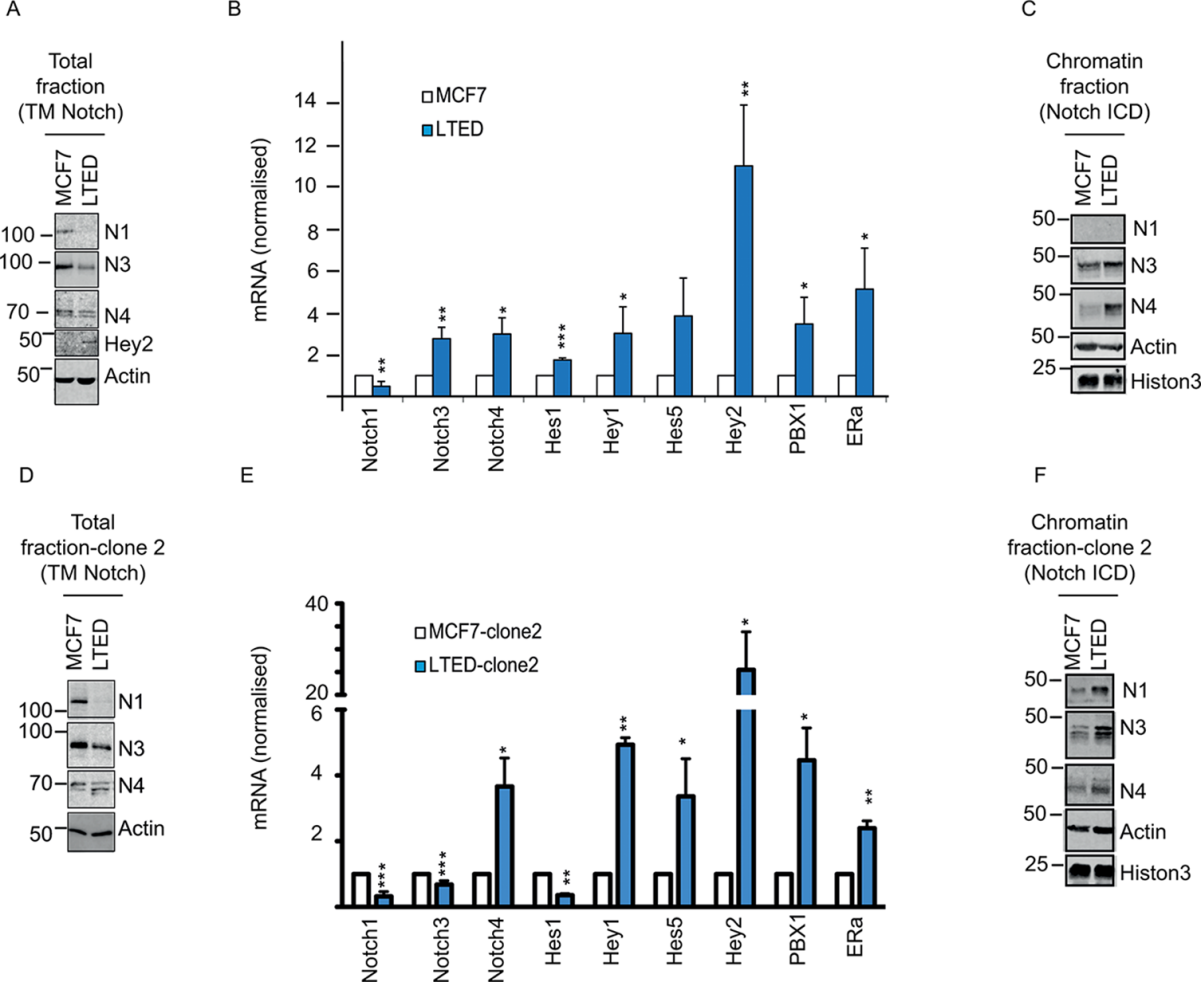
Notch pathway is upregulated in LTED and is dependent on DMXL2 overexpression **A–D.** Representative western blot showing (reported) Notch transmembrane domain (TM) and Notch target Hey2 in MCF7 and LTED cells (clone 1 and 2 respectively) Membranes were blotted with commercially available antibodies against Notch total level **B–E.** q(RT) PCR mRNA normalised to 28S of Notch receptors and Notch targets **C–F.** Cells were fractionated and the chromatin fraction was loaded. Membranes were blotted with in house-developed antibodies against the intracellular domain (Notch ICD).

We have previously shown that Notch signalling plays a significant role in the development of endocrine therapy resistance [18, 19, 29, 30]. Notch signalling is partly dependent on the absolute amounts of Notch receptors present in the cells. However, there is substantial debate on the importance of different Notch receptors in endocrine resistance (reviewed in [31]). We reasoned that the rate of Notch activation, via its cleavage, is equally important to downstream signalling controlling tumor cell growth and survival. To test this, we first quantified total Notch levels using two isogenic cell lines: MCF7 and long-term estrogen deprived (LTED). The latter were derived from MCF7 following one year estrogen withdrawal to mimic endocrine therapy resistant breast cancer [28]. Notably, we find that all the receptors are downregulated at the protein level (Figure 1A). Notch1 is also downregulated at the mRNA level, whereas Notch3 and Notch4 mRNA levels are not (Figure 1B). Paradoxically, LTED cells contain higher protein levels of the Notch target Hey2, a well-characterized estrogen target gene [32] suggesting that receptor levels alone could not accurately predict Notch signalling (Figure 1A). LTED cells have impaired ERα signalling as a consequence of estradiol starvation, thus suggesting that Hey2 may be uniquely regulated by Notch in endocrine therapy resistant cells. Furthermore, we find that numerous additional Notch targets are also transcriptionally upregulated in LTED cells despite a general reduction in Notch receptor (Figure 1B). The discrepancy between total Notch protein levels and Notch target genes might be explained by hyper-activated Notch signalling via increased cleavage rates. To test this hypothesis, we measured Notch ICDs by chromatin fractionation and western blot, using ICD specific antibodies, which were designed and fully characterised in house (Supplementary Figure S1A–1B). Interestingly, we find that the LTED chromatin fraction presents greater enrichment for the Notch3 and Notch4 ICDs (Figure 1C). Importantly, we also screened a second LTED model, derived in a different lab (LTED clone 2) [10]. We confirmed that the total amount of Notch is dowregulated at protein level (Figure 1D) whereas Notch ICDs are upregulated in the chromatin fraction (Figure 1F). We also find Notch target genes are transcriptionally upregulated in the second clone (Figure 1E) thus confirming hyper-activated Notch signalling in endocrine therapy resistant cells.

### DMXL2 is over-expressed in metastatic breast tumors and is epigenetically activated in endocrine therapy resistant (ETR) cell lines

**Figure 2:**
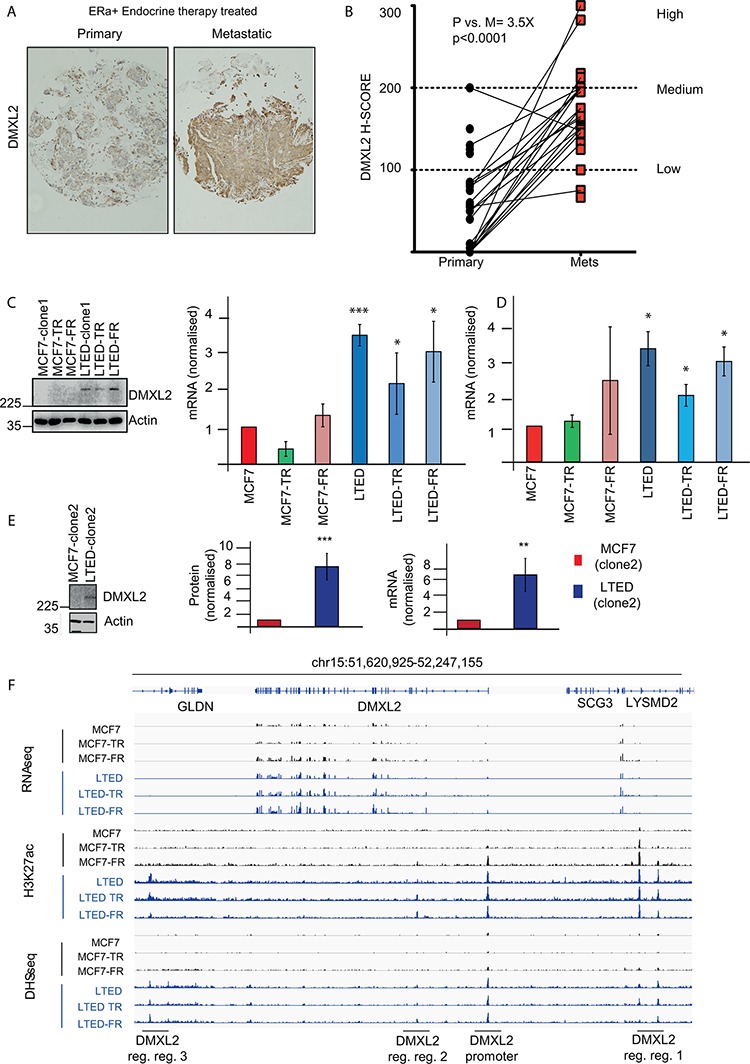
DMXL2 is overexpressed in metastatic tumors resistant to endocrine therapy **A.** Primary and matched Metastatic samples were processed using DMXL2 IHC **B.** H scores were plotted. Pair-wise *T*-test between the average score (3 independent scorers, duplicate sections) was used to establish significance. DMXL2 is overexpressed in ETR cells and it is epigenetically activated. **C.** Representative western blot showing DMXL2 is overexpressed in LTED resistant cells. Graph shows protein normalised to loading control (Actin). q(RT) PCR mRNA levels normalised to 28S. In (C) cells were cultured as described in material and methods. In **D.** cells were cultured in MCF7 media (3 days). (*) *P* < 0.05 (**) *P* < 0.01 (***) *P* < 0.001 **E.** Different clone of MCF7 and LTED showing DMXL2 upregulation. **F.** Snapshots from DNaseI hypersensitivity site (DHS-seq) and Chip-seq for K27ac histone mark near DMXL2 promoter across all 6 breast cancer cell models. Chip-seq tracks display the active regulatory elements near DMXL2 promoter are uniquely found in estrogen deprived cell lines (LTED cells), indicating the activation of DMXL2 gene in LTED cells, but not MCF7 cells.

The recent discovery of DMXL2 as novel Notch regulator, prompted us to look into clinical setting. We built a case set including biopsies from ERα positive breast cancer patients treated with endocrine therapies. For each patient we measured DMXL2 in untreated primary breast cancer samples and matched metastatic samples resistant to endocrine therapy using IHC which revealed that DMXL2 had a membrane-cytoplasmic staining (Figure 2A). Using a pair-wise comparison we found a significant increase in DMXL2 staining in the metastatic samples (Figure 2B), suggesting that DMXL2 is upregulated in endocrine therapy resistant cells *in vivo*. Based on our findings *in vivo*, we decided to explore DMXL2 protein levels using a comprehensive panel of endocrine resistant cells including MCF7 derived tamoxifen or fulvestrant resistant (MCF7-TAMR, MCF7-FULVR), as well as MCF7 cultured in the absence of Estrogen (LTED). In addition, we have analysed two LTED-derived models, which also developed sequential resistance (LTED-TAMR and LTED-FULVR) [28]. Our data indicate that DMXL2 is upregulated in all the LTED models (Figure 2C) while MCF7-TAMR and MCF7-FULVR do not exhibit increased DMXL2 expression suggesting endocrine therapy specific modulation of DMXL2. In addition, we confirmed that changes in DMXL2 expression are not related to different culturing conditions (e.g. chronic drug exposure). LTED maintained high levels of DMXL2 even in the presence of parental MCF7 media (supplemented with E2), thus confirming that DMXL2 is not estradiol dependent but it is epigenetically stably overexpressed (Figure 2D). We also show that DMXL2 is significantly upregulated in the second clone (Figure 2E). DMXL2 overexpression is accompanied by increased chromatin marks typical of active regulatory elements (H3K27ac) at the DMXL2 promoter and nearby putative enhancers (Figure 2F). Similarly, the chromatin near DMXL2 regulatory elements is more accessible to transcription factor regulation in LTED compared to MCF7 lines as shown by DNaseI hypersensitivity assays followed by next generation sequencing. (DHS-seq, Figure 2F). All the following experiments were performed using LTED clone 1 (henceforth identified as LTED).

### DMXL2 depletion impairs notch signalling in endocrine therapy resistant cells

**Figure 3:**
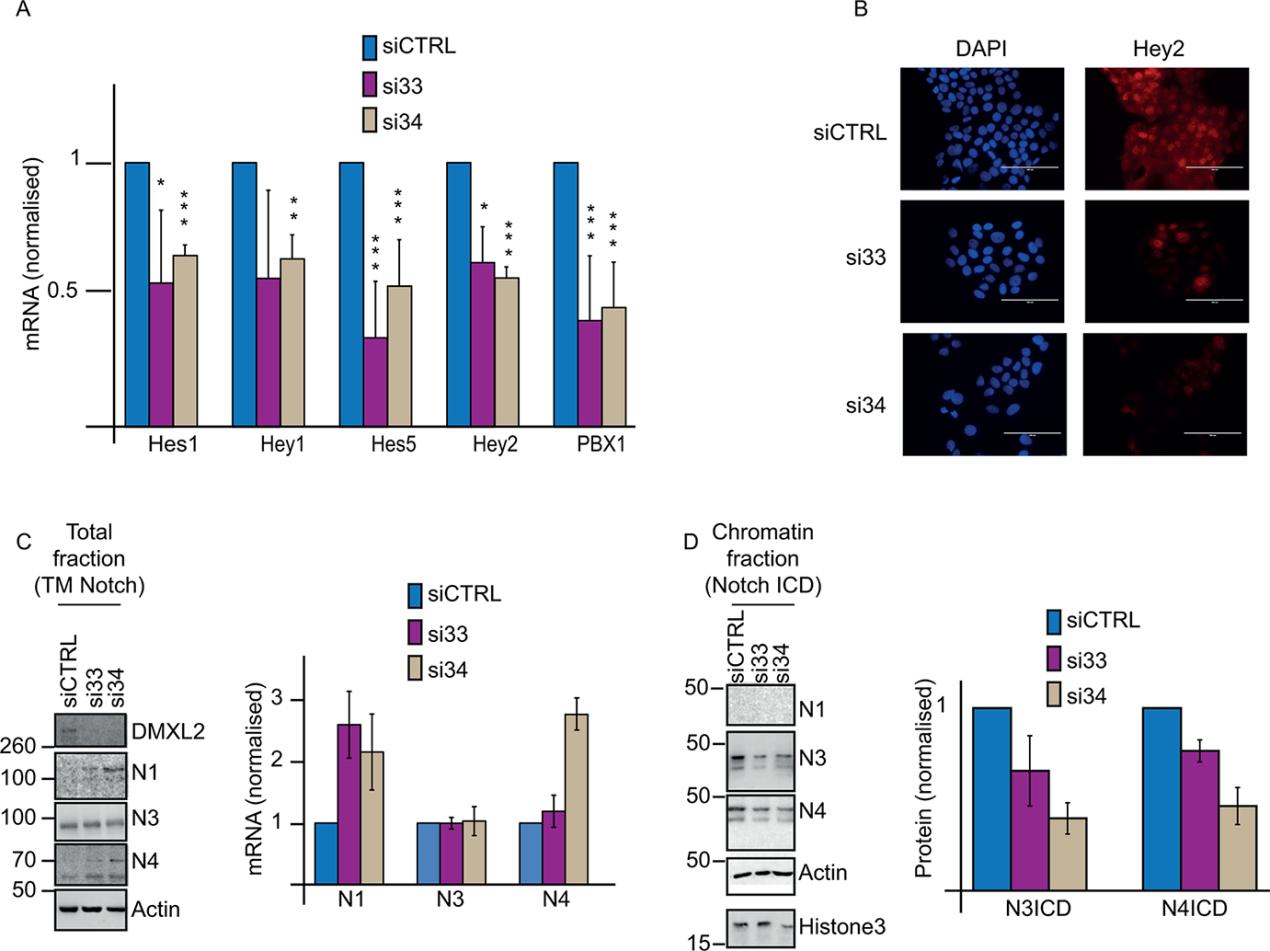
DMXL2 regulates Notch pathway **A.** q(RT) PCR mRNA levels of Notch targets following DMXL2 depletion **B.** Representative immunofluorescence images of Hey2 following siDMXL2 showing Hey2 nuclear signal was impaired. Bars represent 400 uM **C.** Representative western blot showing reported TM Notch accumulates following DMXL2 depletion. Antibodies were used as in 1A **D.** Representative blot showing Notch3 and 4ICD reduced chromatin accumulation following siDMXL2. Antibodies were used as in 2C. Knock down was performed for 48 or 72 hrs (for mRNA or protein extraction respectively). (*) *P* < 0.05 (**) *P* < 0.01 (***) *P* < 0.001. Every experiment is an average of three independent experiments. Bars represent standard deviation.

Previous evidence suggests that DMXL2 might regulate Notch in breast cancer cells and that this regulation is dependent on the interaction between DMXL2 and the V-ATPase pump. [23, 33]. Initially, we postulated that high levels of DMXL2 in endocrine resistant cells lines might result in increased Notch signalling. To test the role of DMXL2 in Notch signalling in the resistant cells, we measured Notch targets following DMXL2 depletion. As expected, siRNA mediated DMXL2 knock-down (Supplementary Figure S2A) significantly impairs Notch downstream signalling (Figure 3A). Furthermore, DMXL2 depleted LTED display less nuclear Hey2 protein levels demonstrating that DMXL2 is required in Notch signalling (Figure 3B). Importantly, following DMXL2 depletion we show augmented trans-membrane (TM) Notch1 and Notch4 at protein and mRNA levels (Figure 3C). Strikingly, this is associated with a decrease in the chromatin bound Notch intracellular domains (NICDs) (Figure 3D). These data strongly suggest that DMXL2 regulates Notch cleavage in endocrine therapy resistant breast cancer cell lines and mediates NICD chromatin accumulation.

### V-ATPase inhibition mimics DMXL2 depletion

**Figure 4:**
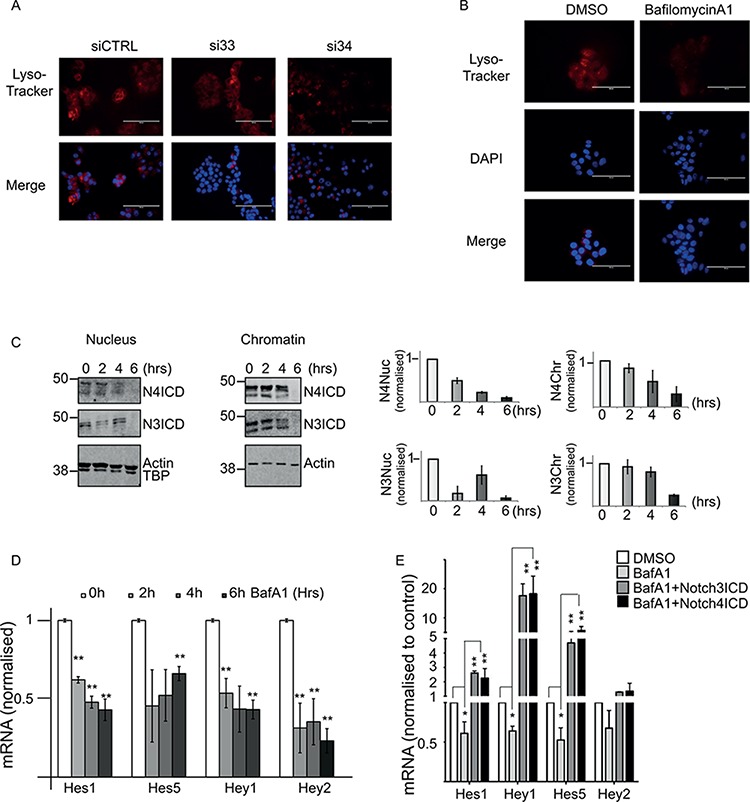
DMXL2 depletion is mirrored by *V-ATPase* inhibition **A.** Representative immunofluorescence showing reduced Lysotracker staining following 72 hours DMXL2 depletion. Bars represent 400 uM **B.** Lysotracker staining of acidic intracellular compartments in LTED cells following Bafilomycin A1 treatment (6 hours). Bars represent 400 uM. **C.** Representative western blot showing Notch ICDs stop accumulating in the nucleus and the chromatin fraction following Bafilomycin A1 (BafA1) treatment in a time dependent manner **D.** q(RT) PCR mRNA of Notch targets levels following BafA1 treatment. In B, C, and D cells were treated with 125 pM of Bafilomycin A1. **E.** Notch targets are rescued by ectopic expression of Notch ICD. Cells were pre-treated for 6 hrs with 500 pM of BafA1 followed by 24 hrs Notch ICD over-expression. Every experiment is an average of three independent experiments. Bars represent standard deviation (*) *P* < 0.05 (**) *P* < 0.01 (***) *P* < 0.001.

DMXL2 has been described as a regulator of the V-ATPase pump in drosophila and mammalian systems. Furthermore, there is a growing body of evidence linking oncogenic Notch signalling to V-ATPase pump in mammals. [23, 34, 35]. First, we wanted to confirm that DMXL2 is required for V-ATPase activity. To do so, we performed DMXL2 depletion and measured pH by Lysotracker staining [36]. As expected, LTED cells depleted for DMXL2 show reduced Lysotracker staining confirming that DMXL2 is a pump regulator (Figure 4A). To directly inhibit the pump, we employed Bafilomycin A1 [37] and measured pH changes in the intracellular compartment. Bafilomycin A1 disrupts V-ATPase function, as shown by the reduction in Lysotracker staining (Figure 4B). These experiments were performed using 125 nM Bafilomycin A1, a dose that does not affect cell proliferation (data not shown). To confirm that Notch signalling is dependent on the V-ATPase activity in endocrine therapy resistant breast cancer cells, we monitored Notch intracellular domain (NICD) accumulation into the chromatin following Bafilomycin A1 treatment. We demonstrate that Notch3 and 4-ICD stop accumulating in the nucleus in a time dependent manner following Bafilomycin A1 treatment (Figure 4C) mirroring the effect of DMXL2 depletion (Figure 3D). In agreement, Notch targets are transcriptionally silenced at the same rate (Figure 4D). To further confirm that DMXL2 is upstream of Notch signalling, we performed a rescue experiment. We used Bafilomycin A1 to down-regulated Notch targets and then over-expressed Notch3 and Notch4ICDs. Notably, we were able to completely rescue their expression (Figure 4E). Overall, these data strongly suggest that increased Notch signalling in LTED is linked to V-ATPase acidification and that this is likely to be mediated by DMXL2 overexpression.

### DMXL2 over-expression correlates with acquisition of EMT phenotype

**Figure 5:**
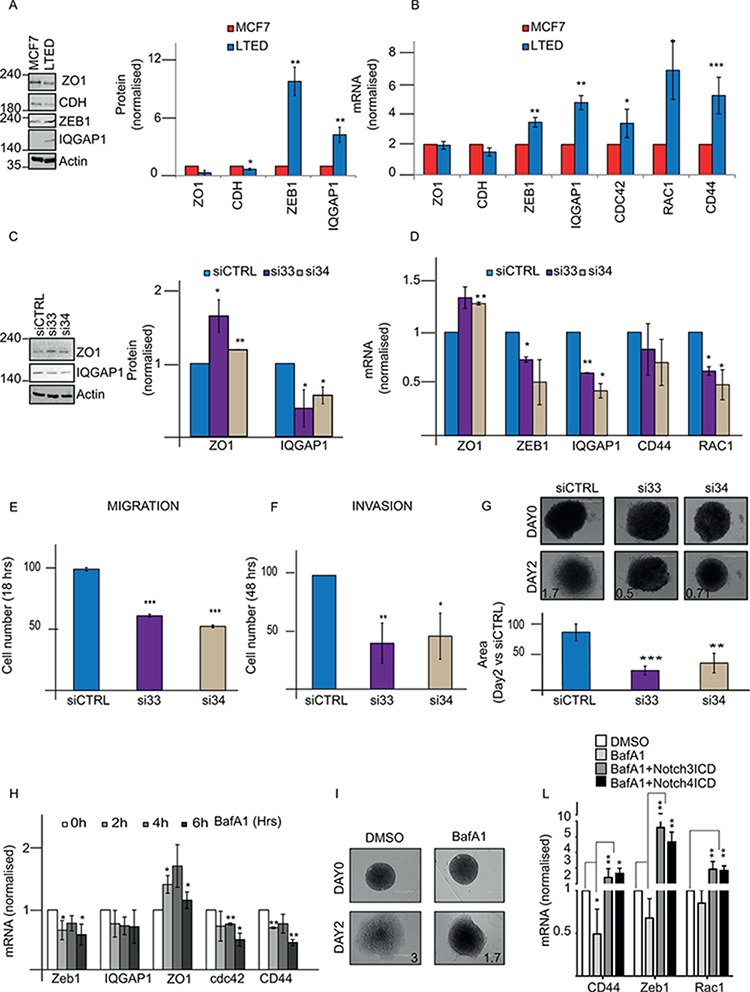
DMXL2 regulates mesenchymal switch in LTED cells **A.** Representative western blot showing EMT targets genes in LTED cells compared to MCF7 **B.** q(RT) PCR mRNA levels of EMT targets normalised to 28S **C.** Representative western blot showing regulation of EMT genes following DMXL2 knock down **D.** q(RT) PCR mRNA of EMT genes after siDMXL2 **E, F.** Knock down was performed on 6 well plate for 24/48 hours. Cells were harvested and counted. 40,000 were seeded on 16 wells ECM plates with/without matrigel (for invasion and migration respectively) and left migrating or invading for 48/24 hours respectively. The graphs represent the difference in cell number compared to siControl **G.** 3D Invasion assay organoids. Representative pictures of organoids embedded in matrigel after 5 days knock down (performed during the hanging time) at day 0 and 2. The graph represents the difference in the area between day 0 (cells just embedded) and day 2 (cells left invading. Bars represent 400 uM. **H.** Bafilomycin A1 impairs cell invasion. Cells were treated as in 4C. qRT-PCR mRNA for EMT genes is shown. **I.** Representative pictures of organoids. Medium containing 500 pM BafA1 was added to the medium for 48 hours after embedding the organoids in matrigel. Day 0 and day 2 images are shown. **L.** EMT targets are rescued by ectopic expression of Notch ICD. Cells were pre-treated for 6 hrs with 500 pM of BafA1 followed by 24 hours Notch ICD over-expression. Every experiment is an average of three independent experiments. Bars represent standard deviation (*) *P* < 0.05 (**) *P* < 0.01 (***) *P* < 0.001.

Within our panel of endocrine therapy specific cells, we noticed that LTED were uniquely associated with migratory and invasive phenotypes (Nguyen *et al*. under revision). Importantly, Notch signalling has been linked to the epithelial to mesenchymal transition (EMT) phenotype [29, 38]. Transcriptional and protein assays confirm that key EMT genes (ZEB1, IQGAP1, CDC42, and CD44) are significantly upregulated in LTED cells at mRNA levels and at protein levels (ZEB1 and IQGAP1). Moreover, loss of E-Cadherin and ZO1, a tight junction marker [39], were downregulated thus confirming LTED cells underwent EMT (Figure 5A–5B). Recently, Tuttle and colleagues proposed that DMXL2 controls migration in Zebrafish Neural Crest [40] through its associated binding partner V-ATPase pump. Therefore we hypothesized that overexpressed DMXL2 could play a role in determining the invasive behaviour of LTED cell lines. DMXL2 depletion leads to a significant downregulation of the majority of EMT genes (IQGAP1 5C, D and ZEB1, CD44, and RAC2 in Figure 5D) whilst tight junction marker ZO1 was upregulated (Figure 5C–5D). Strikingly, DMXL2 depletion also resulted in a significant decrease in real time migration and invasion cells capacity (Figure 5E–5F). These data suggest that DMXL2 is involved in the aggressive phenotype exhibited by LTED cells. Notably, DMXL2 depletion did not affect cell viability (Supplementary Figure S2B). Next, we confirmed that DMXL2 siRNA mediated knock down using a 3D invasion assay. Remarkably, DMXL2 depletion significantly impairs the formation of pseudopodia and the invasion of the extra-cellular matrix in LTED cells (Figure 5G). We then wanted to confirm these findings using Bafilomycin A1 since we have shown that direct inhibition of the V-ATPase pump phenocopy DMXL2 depletion. Interestingly, main EMT targets are significantly reduced upon Bafilomycin A1 treatment and the tight junction marker ZO1 is upregulated (Figure 5H). Furthermore, Bafilomycin A1 treatment significantly impairs invasion in our 3D invasion assay (Figure 5I). Moreover, in agreement with our previous results (Figure 4E) Notch ICD overexpression was sufficient to rescue EMT genes expression in Bafylomicin treated cells (Figure 5L). Altogether, these evidences strongly support DMXL2 as a major player in determining mesenchymal switch in our endocrine resistant cell line model.

### DXML2 has a potential extracellular domain

**Figure 6:**
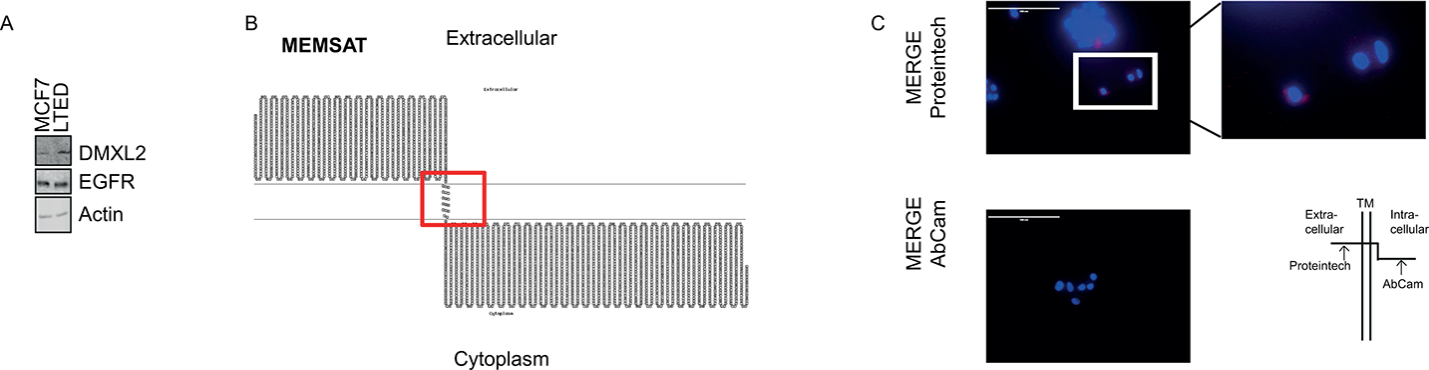
DMXL2 has a potential extracellular domain **A.** Western blot showing cells labeled with biotin and processed as described in material and methods. EGFR and Actin are used as controls **B.** Graphs showing DMXL2 putative transmembrane domain **C.** Representative live immunofluorescence images showing DMXL2 is detected only with the antibody interacting with the potential extracellular domain (AbCAm antibody). Bars represent 400 uM.

The V-ATPase pump complex consists of two different domains of which one is a peripheral membrane sub-complex. DXML2 is thought to control the modulation of the pump assembly/disassembly process [41, 42]. We speculated that DMXL2 could also be a peripheral membrane protein. To test this, we first employed EZ-Link Sulfo-NHS-LC-LC-Biotin to label cell surface proteins and confirm an increase of signal from DMXL2 protein outside LTED cells membrane (Figure 6A). Next, we used several publicly available informatics predictive algorithms (e.g. MEMSAT, PSIPRED, TMHMM, TMPRED [43–46] to test if DMXL2 carries potential transmembrane domains. Notably, several algorithms predicted the same potential transmemembrane domain (Figure 6B and Supplementary Figure S3). We further investigated this possibility, using two different antibodies: one mapping onto the potential outer domain, and one mapping onto the putative cytoplasmic end (They were both fully characterised in Supplementary Figure S2A–2D). Live immunofluorescence without cell permeabilization shows that only the antibody interacting with the potential extra-cellular domain could detect DMXL2 (Figure 6C). Overall, these data strongly suggest that DMXL2 could be a peripheral membrane protein with a potentially targetable extracellular domain.

## DISCUSSION

Here we demonstrate that DMXL2 overexpression drives Notch signalling and EMT transition in endocrine therapy resistant breast cancer cells. DMXL2 activity impinges on the V-ATPase pump and can be antagonized using siRNA or chemical inhibitors. More importantly, DMXL2 increases *in vivo* in patients who relapse following treatment with endocrine therapy.

Notch is involved in numerous pathways and plays a significant role in tumor progression and metastasis [10]. However, there is still a lack of understanding of the molecular details of Notch signalling, especially in cancer cells. On this ground, we investigated DMXL2 as it was recently discovered as a novel potential Notch regulator [22, 23]. We show that DMXL2 expression significantly correlates with the development of endocrine resistance. However we cannot exclude that a resistant pre-existent population with high levels of DMXL2 emerge during chronic exposure to treatment. At any rate our data provide novel insights that explain apparent contradictory results such as that ETR cells have enhanced Notch signalling while having lower total Notch receptors. This suggests the processing rate of the Notch receptors cleavage might be more accurate in predicting actual Notch signalling compared the total unprocessed protein levels. DMXL2 appears to be essential in this process as direct DMXL2 suppression or blockage of the V-ATPase activity result in an important reduction of NICD accumulation in the chromatin fraction.

Drug resistance is a major hurdle for patients with ERα breast cancer. A spectrum of published research supports Notch as a key player in drug resistance [18, 19, 29, 47]; therefore targeting this pathway is becoming more and more attractive [48, 49]. The mechanisms behind drug resistance are not completely understood but increasing evidence suggests that resistant cells may epigenetically activate alternative pathways [19, 50]. Our model suggests that DMXL2 is upregulated in response to endocrine therapy treatment and contributes to a the more aggressive phenotype observed in these cells.

A recent work from Tuttle and colleagues uncovered a potential role of DMXL2 and V-ATPase in EMT/migration of neuronal crest in Zebrafish models. Based on these findings and on the highlight of our recent work [29], we wanted to explore the role of DMXL2 in driving the mesenchymal switch in therapy resistant breast cancer. We reveal for the first time that DMXL2 is responsible for driving the mesenchymal switch in ETR cells and that blocking DMXL2 significantly impairs both migration and invasion. We also demonstrate that DMXL2 is a V-ATPase interacting partner and the pump acidification controls Notch signalling. Indeed, blocking V-ATPase using Bafilomicyn A1 mirrors DMXL2 depletion and impairs Notch binding to the chromatin as well as invasion. DMXL2 overexpression in ETR cell lines, and its implication in metastatic development, gave us the idea of looking in the clinical setting. To our knowledge, this is the first report indicating that DMXL2 protein is present in patients with ERα breast cancer. Notably, DMXL2 increases dramatically during breast cancer progression, in agreement with our cell line model.

V-ATPase inhibition, through Bafilomycin A1, causes tumor regression and metastasis inhibition *in vivo* and *in vitro* [51]. Based on these facts, we reasoned that manipulating Notch via DMXL2 inhibition could be an exciting route that warrants further investigation. At the moment, the approach taken to target Notch signalling had been through the use of GSIs that inhibit signalling by all four Notch receptors [52, 53]. Our recent works showed that using monoclonal antibodies to target the extra-cellular domain of Nicastrin, the catalytic subunit of the GS complex, greatly reduced the tumor bulk *in vivo* and limits invasion and migration *in vitro* [29, 54]. Here, we show that DMXL2 likely has an extracellular portion. This warrants further investigation focusing on the development of monoclonal antibodies targeting DMXL2 as an alternative option to block Notch in breast cancer.

## MATERIALS AND METHODS

### Cell culture

Cells were originated as previously described [28]. MCF7 cells were maintained in Dulbecco's modified Eagle's medium (DMEM) containing 10% fetal calf serum (FCS). LTED were maintained in phenol-red free DMEM containing 10% charcoal stripped fetal calf serum (SFCS). Both media were supplemented with 2 mM L-glutamine, 100 units/mL penicillin, 0.1 mg/mL. 10^−8^ M Estradiol (E2758 Sigma) was added routinely to MCF7. Cells used in Figures 1 and 2 and named “clone 2”, were independently derived [19]. 10^−7^ Tamoxifen (H7904 Sigma) and Fulvestrant (I4409 Sigma) were added to TAMR and FULVR cells.

### Migration and invasion assay

This assay was performed as previously described (Nguyen et al., under revision). Briefly, real time cell analysis (RTCA) was carried on using xCELLigence technology (Cambridge Bioscience), and 40,000 cells were seeded on a well of 16-transwell CIM-PLATES with/without matrigel to monitor migration and invasion respectively. Prior to each experiment, cells were subjected to 48/24 hrs DMXL2 knock down, harvested, counted, and then allowed to migrate or invade for 24–48 hours respectively (total knock down 72 hrs). Serum free medium was used to resuspend and seed the cells, and 10% FCS or DC/FCS full medium was used as chemo-attractant.

### RNA extraction and real time RT_PCR

RNA extraction and cDNA preparation were performed as previously described [29]. Briefly, QIAGEN columns were used for RNA extraction. 2ug were retro-transcribed using High Capacity cDNA Reverse Transcription (AB 4368814 Applied Biosystem). 2X SYBR GREEN mix (4309155 Invitrogen) was used to perform quantitative real time RT-PCR. The PCR amplification was as follows: 95°C (10 min), 95°C (10 sec), 60°C (30 sec), 72°C (30 seconds), 40 cycles followed by melting curve analysis. Melting curves were analysed after 40 cycles. Expression levels of each gene were evaluated using comparative threshold cycle (Ct) method using the 2-ΔΔCt method with normalization to 28S housekeeping gene. Primers sequences are available upon request.

### siRNA

Silencers Select Small interfering RNA (siRNA) against DMXL2 were purchased from Invitrogen (s23533, s23534). Silencer Select Negative 1 was used as control (4404020). Briefly, 300,000 cells were seeded on a 6 well plate. The following day Lipofectamine 2000 (11668) was used according to the manufacturer protocol. 5nM siRNA were used as final concentration. RNA and proteins were analysed 24 and 72 hours later respectively.

### Overexpression

Notch1, Notch3 and Notch4 ICDs were a kind gift from Keith Brennan. Plasmids were fully sequenced. Lipofectamine 2000 was used according to the instructions. 300,000 cells were seeded on day 1. The following day, 1 ug of DNA was used for transfection. Cells were harvested and lysed 24 hrs later.

### Rescue experiment

LTED cells (200,000) were seeded on 6 well plates. The following day 500pM Bafilomycin A1 was added to the medium. After 6 hours, cells were transfected with 1 ug of Notch3, 4 plasmid using Lipofectamine 2000 according to the manufacturer instructions. 24 hours later, mRNA was extracted according to the manufacturer instructions (QIAGEN). DMSO was used as control.

### 3D organoids assay (hanging drop assay)

A 3D assay was performed as previously described (Nguyen et al. under revision). Briefly, 250,000 cells were resuspended in 1ml of medium. Drops (20 ul) were placed onto the lid of a 10 cm dish, then the lid was flipped onto of the dish, containing 5 ml of media to prevent evaporation. The dish was then incubated for 5 days at 37°C. On day 5, the newly formed organoids were mounted on 10ul phenol red free matrigel (356237) on a coverslip in a 24 well plate. Matrigel was left for 1 hr at 37°C in the incubator to solidify, then 500 ul of full medium was added. Pictures were taken at day 0 and day 2. In knock down experiments, siDMXL2 or siControl were added to the cells before making the drops. In Bafilomycin A1 experiments, the drug was added to the medium on day 0 for 48 hours.

### Immunofluorescence

Briefly, cells were seeded on coverslips on a 6 well plate. On the final day, cells were washed twice with room temperature PBS and 4% PFA/PBS was added for 15 minutes. Cells were washed twice with PBS and NH4Cl was added as quencher. 1% Triton/PBS was added for 5 minutes. 10% BSA/PBS was used as blocking reagent. 5% BSA/PBS was used to dilute antibodies and Alexa-fluor 488 or 594labelled anti-rabbit or anti-mouse secondary antibodies. Cells were mounted in mounting medium (MOWIOL) containing 4′,6-diamidino-2-phenylindole. For live immunofluorescence, no fixative was used. Pictures were acquired using EVOS microscope system (Advanced Microscopy Group, Bothell, WA, USA) or Leica SP5 confocal microscope.

### Antibodies-drugs

We used two antibodies against DMXL2 (24415-1-AP from Proteintech) for western blot and immunofluorescence and ab122552 (abCam) for IHC and immunofluorescence. Antibodies against Notch1 and Notch3 were purchased from Cell Signalling (3608, and 2889) and Notch4 was purchased from Santa Cruz (Sc-5594 H-225). Actin and V5 antibodies were purchased from abCam (ab6276 and ab9116). ZEB1 (3396), ZO1 (8193), E-Cadherin (3195), and IQGAP1 (2293) antibodies were purchased from Cell Signalling. Bafilomycin A1 was purchased from Sigma (B1793). TBP was purchased from Santa Cruz. LysoTracker (LysoTracker® Red DND-99 Life Technologies) was used according to the manufacturer protocol for labelling acidic organelles in live cells following Bafilomycin A1 and DMXL2 siRNA treatments. In knock down experiments, the LysoTracker was added after 72 hours. Estradiol for culturing MCF7 was also purchased from Sigma and diluted in 100% Ethanol in a final concentration of 10^−^7M. Biotinylation assay was performed according to the manufacturer protocol (21435 Thermo Scientific). GSI was purchased from Pfizer, Inc (New York, NY, USA) (PF03084014). Dll4 ligand was purchased from abCAm (108557).

### Western blot and fractionation

RIPA (Sigma) was used to prepare total lysate. Fractionation method was performed as previously described [55]. Bicinchoninic Acid (BCA) assay (Thermo Scientific) was used to determine protein concentration. Equal amounts of lysates were subjected to immunoblotting on SDS-PAGE. IRDyes IR680, IR680-LT, or IR800 (LI-COR) were used as secondary antibodies. LI-COR Odyssey 2.1 system was used to visualise proteins. 16-bit images were analyzed and quantified using Image J.

### Tissue microarray (TMA) of paired primary and secondary breast cancers

Twenty primary breast carcinomas with a paired metastasis were acquired from the pathology archives of Charing Cross Hospital, London, UK. A tissue microarray was constructed using a manual microarray (Beecher) and 0.6 mm punches. The tissue microarray was immunohistochemically profiled for DMXL2 and other biological antibodies as previously described (ref ?). DMXL2 rabbit antibody (ab122552, abCam) was optimized to a working concentration (1:500 in PBS); utilizing tissue sections (5 μm). Antigen retrieval was performed using 0.01M citrate buffer, pH 6.0 followed by blocking in 0.3% hydrogen peroxide in PBS, then in normal goat serum (20 μl per ml) for 30 min. The primary antibody was incubated overnight at 4^°^C, and then detected using anti-rabbit secondary antibody (Vector Laboratories), Vectastain Elite peroxidase ABC kit, and ImmPACT DAB kit (Vector Laboratories). Subsequently, 4 μm TMA sections were immuno-stained using the optimized staining protocol, including negative controls (omission of the primary antibody). Staining was scored based on the H-score and Allred Quick score (LM, MF). Cores were scored using an H-score by three independent investigators (including one consultant pathologist) blinded to the clinico-pathological characteristics of patients. H = (3 × % of strongly stained cells) + (2 × % of moderately stained cells) + (1 × % of weakly stained cells) + (0 × % of cells without staining). This gave a score ranging from total absence of DMXL2 in the tumor compartment (H-score 0) to DMXL2 expression in tumor cells equivalent to surrounding normal and stromal cells (H-score 300).

### Statistical analysis

Two-tailed paired *T*-test was used to generate *P*-values (indicated as *P = 0.05, **P = 0.01, and ***P = 0.001). Anova test with Tukey's post test was used to generate *P*-values for the rescue experiment (*P = 0.05, **P = 0.01).

## SUPPLEMENTARY FIGURES



## References

[1] Stylianou S, Clarke RB, Brennan K (2006). Aberrant activation of notch signaling in human breast cancer. Cancer research.

[2] Andersson ER, Lendahl U (2014). Therapeutic modulation of Notch signalling--are we there yet?. Nature reviews Drug discovery.

[3] Kandachar V, Roegiers F (2012). Endocytosis and control of Notch signaling. Current opinion in cell biology.

[4] Le Borgne R (2006). Regulation of Notch signalling by endocytosis and endosomal sorting. Current opinion in cell biology.

[5] Tolcher AW, Chugh R, Chambers G, Thorpe V, Dupont J, Hill D (2012). A first-in-human phase I study to evaluate the fully human monoclonal antibody OMP-59R5 (anti-Notch2/3) administered intravenously to patients with advanced solid tumors. Journal of Clinical Oncology.

[6] Doody RS, Raman R, Farlow M, Iwatsubo T, Vellas B, Joffe S (2013). A phase 3 trial of semagacestat for treatment of Alzheimer's disease. The New England journal of medicine.

[7] Ntziachristos P, Lim JS, Sage J, Aifantis I (2014). From fly wings to targeted cancer therapies: a centennial for notch signaling. Cancer cell.

[8] Fan X, Khaki L, Zhu TS, Soules ME, Talsma CE, Gul N (2010). NOTCH pathway blockade depletes CD133-positive glioblastoma cells and inhibits growth of tumor neurospheres and xenografts. Stem cells.

[9] Giachino C, Taylor V (2014). Notching up neural stem cell homogeneity in homeostasis and disease. Frontiers in neuroscience.

[10] Li Y, Ma J, Qian X, Wu Q, Xia J, Miele L (2013). Regulation of EMT by Notch signaling pathway in tumor progression. Current cancer drug targets.

[11] Espinoza I, Pochampally R, Xing F, Watabe K, Miele L (2013). Notch signaling: targeting cancer stem cells and epithelial-to-mesenchymal transition. OncoTargets and therapy.

[12] Ginnebaugh KR, Ahmad A, Sarkar FH (2014). The therapeutic potential of targeting the epithelial-mesenchymal transition in cancer. Expert opinion on therapeutic targets.

[13] Artavanis-Tsakonas S, Rand MD, Lake RJ (1999). Notch signaling: cell fate control and signal integration in development. Science.

[14] Bolos V, Mira E, Martinez-Poveda B, Luxan G, Canamero M, Martinez C (2013). Notch activation stimulates migration of breast cancer cells and promotes tumor growth. Breast Cancer Research.

[15] Donepudi MS, Kondapalli K, Amos SJ, Venkanteshan P (2014). Breast cancer statistics and markers. Journal of cancer research and therapeutics.

[16] Musgrove EA, Sutherland RL (2009). Biological determinants of endocrine resistance in breast cancer. Nature reviews Cancer.

[17] Osborne CK, Schiff R (2011). Mechanisms of endocrine resistance in breast cancer. Annual review of medicine.

[18] Austreid E, Lonning PE, Eikesdal HP (2014). The emergence of targeted drugs in breast cancer to prevent resistance to endocrine treatment and chemotherapy. Expert opinion on pharmacotherapy.

[19] Magnani L, Stoeck A, Zhang X, Lanczky A, Mirabella AC, Wang TL (2013). Genome-wide reprogramming of the chromatin landscape underlies endocrine therapy resistance in breast cancer. Proceedings of the National Academy of Sciences of the United States of America.

[20] Magnani L, Brunelle M, Gevry N, Lupien M (2012). Chromatin landscape and endocrine response in breast cancer. Epigenomics.

[21] Ji ZL, Mohammed H, Webber A, Ridsdale J, Han N, Carroll JS (2014). The forkhead transcription factor FOXK2 acts as a chromatin targeting factor for the BAP1-containing histone deubiquitinase complex. Nucleic Acids Research.

[22] Yan Y, Denef N, Schupbach T (2009). The Vacuolar Proton Pump, V-ATPase, Is Required for Notch Signaling and Endosomal Trafficking in Drosophila. Developmental Cell.

[23] Sethi N, Yan Y, Quek D, Schupbach T, Kang YB (2010). Rabconnectin-3 Is a Functional Regulator of Mammalian Notch Signaling. The Journal of Biological Chemistry.

[24] Forgac M (2007). Vacuolar ATPases: rotary proton pumps in physiology and pathophysiology. Nature reviews Molecular cell biology.

[25] Santen R, Jeng MH, Wang JP, Song R, Masamura S, McPherson R (2001). Adaptive hypersensitivity to estradiol: potential mechanism for secondary hormonal responses in breast cancer patients. The Journal of Steroid Biochemistry.

[26] Brodie A, Jelovac D, Sabnis G, Long B, Macedo L, Goloubeva O (2005). Model systems: Mechanisms involved in the loss of sensitivity to letrozole. The Journal of Steroid Biochemistry.

[27] Martin LA, Ghazoui Z, Weigel MT, Pancholi S, Dunbier A, Johnston S (2011). An *in vitro* model showing adaptation to long-term oestrogen deprivation highlights the clinical potential for targeting kinase pathways in combination with aromatase inhibition. Steroids.

[28] Shaw LE, Sadler AJ, Pugazhendhi D, Darbre PD (2006). Changes in oestrogen receptor-alpha and -beta during progression to acquired resistance to tamoxifen and fulvestrant (Faslodex, ICI 182,780) in MCF7 human breast cancer cells. The Journal of steroid biochemistry and molecular biology.

[29] Lombardo Y, Faronato M, Filipovic A, Vircillo V, Magnani L, Coombes RC (2014). Nicastrin and Notch4 drive endocrine therapy resistance and epithelial to mesenchymal transition in MCF7 breast cancer cells. Breast cancer research: BCR.

[30] Zheng LH, Zhao YH, Feng HL, Liu YJ (2014). Endocrine resistance in breast cancer. Climacteric: the journal of the International Menopause Society.

[31] Espinoza I, Miele L (2013). Notch inhibitors for cancer treatment. Pharmacology & therapeutics.

[32] Bourdeau V, Deschenes J, Laperriere D, Aid M, White JH, Mader S (2008). Mechanisms of primary and secondary estrogen target gene regulation in breast cancer cells. Nucleic Acids Research.

[33] Einhorn Z, Trapani JG, Liu Q, Nicolson T (2012). Rabconnectin3alpha promotes stable activity of the H+ pump on synaptic vesicles in hair cells. The Journal of neuroscience: the official journal of the Society for Neuroscience.

[34] Kobia F, Duchi S, Deflorian G, Vaccari T (2014). Pharmacologic inhibition of vacuolar H+ ATPase reduces physiologic and oncogenic Notch signaling. Molecular oncology.

[35] Rath S, Liebl J, Furst R, Vollmar AM, Zahler S (2014). Regulation of endothelial signaling and migration by v-ATPase. Angiogenesis.

[36] Chazotte B (2011). Labeling lysosomes in live cells with LysoTracker. Cold Spring Harbor protocols.

[37] Huss M, Wieczorek H (2009). Inhibitors of V-ATPases: old and new players. The Journal of experimental biology.

[38] Gonzalez DM, Medici D (2014). Signaling mechanisms of the epithelial-mesenchymal transition. Science signaling.

[39] Gumbiner B, Lowenkopf T, Apatira D (1991). Identification of a 160-Kda Polypeptide That Binds to the Tight Junction Protein-Zo-1. Proceedings of the National Academy of Sciences of the United States of America.

[40] Tuttle AM, Hoffman TL, Schilling TF (2014). Rabconnectin-3a regulates vesicle endocytosis and canonical Wnt signaling in zebrafish neural crest migration. PLoS biology.

[41] Kane PM (2012). Targeting Reversible Disassembly as a Mechanism of Controlling V-ATPase Activity. Current Protein & Peptide Science.

[42] Tata B, Huijbregts L, Jacquier S, Csaba Z, Genin E, Meyer V (2014). Haploinsufficiency of Dmxl2, encoding a synaptic protein, causes infertility associated with a loss of GnRH neurons in mouse. PLoS biology.

[43] Jones DT (2007). Improving the accuracy of transmembrane protein topology prediction using evolutionary information. Bioinformatics.

[44] Buchan DW, Minneci F, Nugent TC, Bryson K, Jones DT (2013). Scalable web services for the PSIPRED Protein Analysis Workbench. Nucleic Acids Research.

[45] Jones DT (1999). Protein secondary structure prediction based on position-specific scoring matrices. Journal of molecular biology.

[46] Moller S, Croning MDR, Apweiler R (2001). Evaluation of methods for the prediction of membrane spanning regions. Bioinformatics.

[47] Hsu KT, Yu XM, Audhya AW, Jaume JC, Lloyd RV, Miyamoto S (2014). Novel Approaches in Anaplastic Thyroid Cancer Therapy. The oncologist.

[48] McAuliffe SM, Morgan SL, Wyant GA, Tran LT, Muto KW, Chen YS (2012). Targeting Notch, a key pathway for ovarian cancer stem cells, sensitizes tumors to platinum therapy. Proceedings of the National Academy of Sciences of the United States of America.

[49] Liu JT, Mao ZF, Huang J, Xie SP, Liu TS, Mao ZF (2014). Blocking the NOTCH pathway can inhibit the growth of CD133-positive A549 cells and sensitize to chemotherapy. Biochemical and Biophysical Research Communications.

[50] Yashiro-Ohtani Y, Wang H, Zang C, Arnett KL, Bailis W, Ho Y (2014). Long-range enhancer activity determines Myc sensitivity to Notch inhibitors in T cell leukemia. Proceedings of the National Academy of Sciences of the United States of America.

[51] Graham RM, Thompson JW, Webster KA (2014). Inhibition of the vacuolar ATPase induces Bnip3-dependent death of cancer cells and a reduction in tumor burden and metastasis. Oncotarget.

[52] Wei P, Walls M, Qiu M, Ding R, Denlinger RH, Wong A (2010). Evaluation of selective gamma-secretase inhibitor PF-03084014 for its antitumor efficacy and gastrointestinal safety to guide optimal clinical trial design. Molecular cancer therapeutics.

[53] Fouladi M, Stewart CF, Olson J, Wagner LM, Onar-Thomas A, Kocak M (2011). Phase I trial of MK-0752 in children with refractory CNS malignancies: a pediatric brain tumor consortium study. Journal of clinical oncology: official journal of the American Society of Clinical Oncology.

[54] Filipovic A, Lombardo Y, Faronato M, Abrahams J, Aboagye E, Nguyen QD (2014). Erratum to: Anti-nicastrin monoclonal antibodies elicit pleiotropic anti-tumour pharmacological effects in invasive breast cancer cells. Breast cancer research and treatment.

[55] Faronato M, Patel V, Darling S, Dearden L, Clague MJ, Urbe S (2013). The deubiquitylase USP15 stabilizes newly synthesized REST and rescues its expression at mitotic exit. Cell cycle.

