# Supplementary material for: DMXL2 drives epithelial to mesenchymal transition in hormonal therapy resistant breast cancer through notch hyper-activation
Source: Oncotarget. 2015 Jun 4;6(26):22467–79. doi: 10.18632/oncotarget.4164 (PMC4673176; doi:10.18632/oncotarget.4164)
Supplement: Supplementary file 1 [file oncotarget-06-22467-s001.pdf]

SUPPLEMENTARY FIGURES

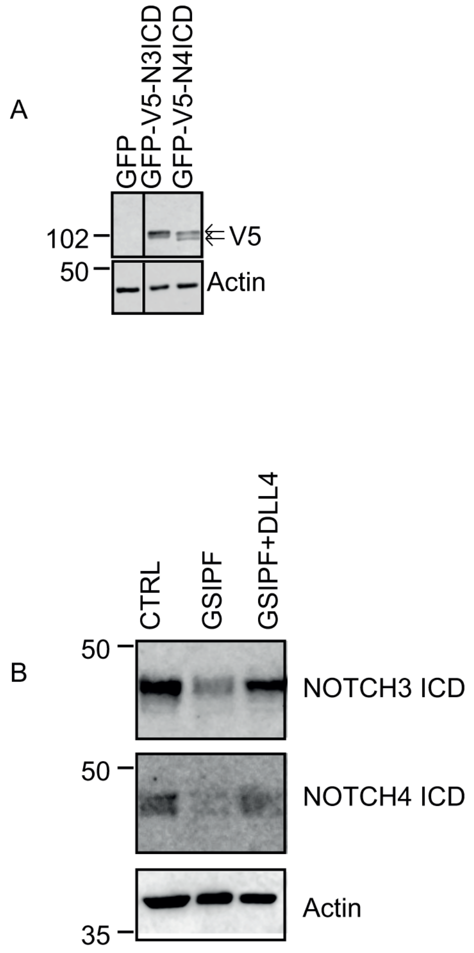

Supplementary Figure S1: A. Characterization of Notch antibodies targeting intracellular domain. Notch3 and 4 intracellular domains (NICDs) were overexpressed for 24 hours in LTED cells. Cells were lysed using RIPA buffer. The constructs contain both GFP and V5. V5 antibody was used to determine NotchICD molecular weight. (GFP+V5+NotchICD = 27+24+48 KDa = ~110 kDa) Vector containing GFP was transfected as control. **B.** MCF7 cells were treated with 10 uM GSI PF03084014 for 72 hours. Cells were rinsed twice in PBS and DLL4 ligand was added to the medium for 1 hour. Cells were then fractionated and chromatin fraction was loaded on a gel. Actin was used as loading control.

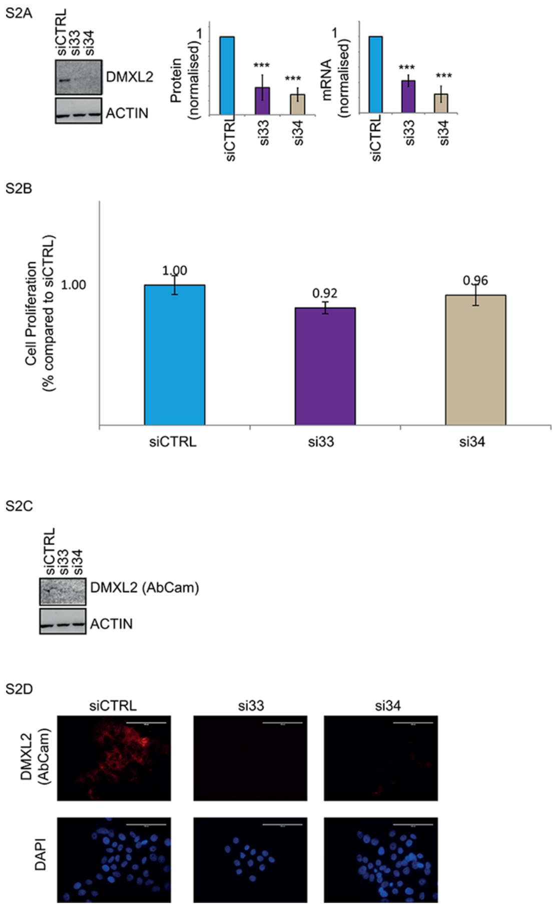

Supplementary Figure S2: DMXL2 antibodies validation A. Two different siRNAs were used to validate DMXL2 knock down. Graphs show protein and mRNA normalised to Actin and 28S respectively. **B.** DMXL2 knock down does not affect proliferation **C–D.** Western blot and representative immunofluorescence showing validation of abCam antibody. This antibody maps on the putative intracellular domain. Bars represent 400 uM.

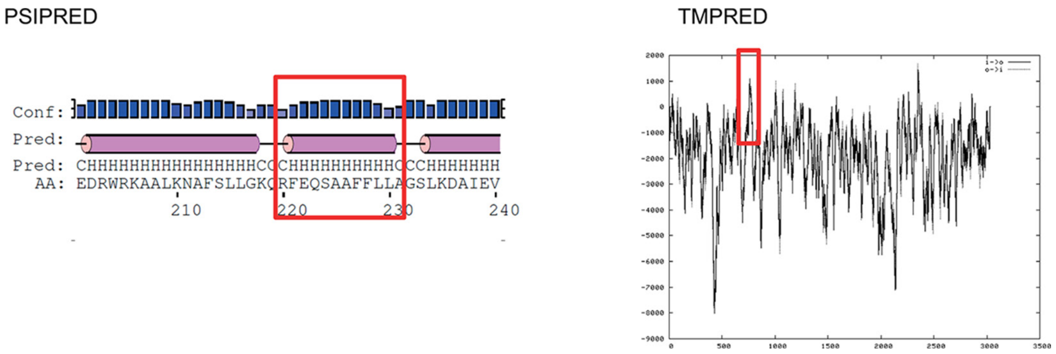

Supplementary Figure S3: Graphs showing DMXL2 transmembrane domain predicted by different algorithms.
